# P-1156. Procalcitonin Assay in Predicting Etiology in Young Febrile Infants Visiting the Emergency Department in Korea

**DOI:** 10.1093/ofid/ofae631.1342

**Published:** 2025-01-29

**Authors:** Haemin Kim, Min Young Kim, Jee Yeon Baek, Ji Young Lee, Seo Hee Yoon, Heeyeon Kim, Ji-Man Kang

**Affiliations:** Department of Pediatrics, Severance Children’s Hospital, Yonsei University College of Medicine, Seoul, South Korea., Goyang, Kyonggi-do, Republic of Korea; Severance Children’s Hospital, Yonsei University College of Medicine, Seoul, Seoul-t'ukpyolsi, Republic of Korea; Yonsei University College of Medicine, Seoul, Seoul-t'ukpyolsi, Republic of Korea; Yonsei University College of Medicine, Seoul, Seoul-t'ukpyolsi, Republic of Korea; Department of Pediatrics, Severance Children’s Hospital, Yonsei University College of Medicine, Seoul, South Korea., Goyang, Kyonggi-do, Republic of Korea; Department of Pediatrics, Severance Children’s Hospital, Yonsei University College of Medicine, Seoul, South Korea., Goyang, Kyonggi-do, Republic of Korea; Severance Children’s Hospital, Yonsei University College of Medicine, Seoul, Seoul-t'ukpyolsi, Republic of Korea

## Abstract

**Background:**

There have been no studies conducted in Korea on the role of procalcitonin as a predictive factor for serious bacterial infection (SBI) in young febrile infants under 90 days of age, and it is not included in insurance reimbursement criteria despite its potential clinical utility. Our aim was to assess the role of the procalcitonin assay in distinguishing the etiology of fever, including SBI and simple urinary tract infection (sUTI), in young febrile infants in Korea.

The sensitivity and the specificity of procalcitonin (≥0.5 ng/mL) for SBI and sUTI

**Figure:**
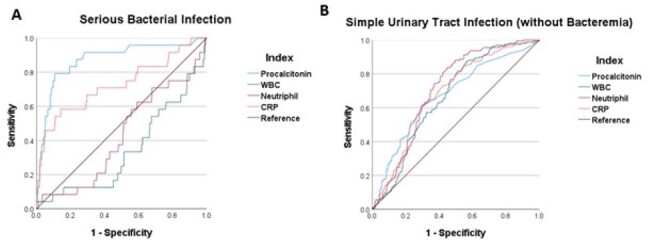

A. The sensitivity and the specificity of procalcitonin for SBI

B. The sensitivity of the specificity procalcitonin for sUTI

**Methods:**

This retrospective study was conducted on patients under 90 days of age who visited the pediatric emergency department (ED) at Severance Children's Hospital and underwent procalcitonin assay from January 2019 to February 2024. Patients who visited the ED for reasons other than fever or were under 29 days of age were excluded. The SBIs were defined as cases of the identification of pathogens in sterile fluids such as blood and cerebrospinal fluid, while sUTIs were defined as UTIs without bacteremia. Viral infections were defined as cases where a virus was identified through testing or when symptoms or contact history clearly indicated viral infection; otherwise, they were classified as unknown. The cut-off value for procalcitonin was set at 0.5 ng/mL.

**Results:**

A total of 633 patients were collected, of whom 131 were excluded, resulting in 502 individuals included in the analysis. Among them, half were aged between 29-60 days and the other half between 61-90 days, with six admissions to the intensive care unit and no fatalities. By etiology, there were 22 cases of SBI (4.4%), 161 cases of sUTI (32.1%), 147 cases of viral infection (29.3%), and 172 cases classified as unknown (34.3%). The median values of procalcitonin were 5.03 (range, 0.08-100) for SBI, 0.35 (range, 0.05-52.2) for sUTI, 0.13 (range, 0.04-53.3) for viral infection, and 0.15 (range, 0.04-100) for unknown. The sensitivity of procalcitonin (≥0.5 ng/mL) for SBI and sUTI was 81.8% and 42.2% respectively, while the specificity was 77.8% and 88.3% respectively. (see Figure)

**Conclusion:**

In febrile young infants in Korea, the utility of procalcitonin as a predictor of SBI is significant, warranting consideration for its proactive integration into clinical practice.

**Disclosures:**

**All Authors**: No reported disclosures

